# RORγt inhibitors block both IL-17 and IL-22 conferring a potential advantage over anti-IL-17 alone to treat severe asthma

**DOI:** 10.1186/s12931-021-01743-7

**Published:** 2021-05-22

**Authors:** David Lamb, Dorothy De Sousa, Karsten Quast, Katrin Fundel-Clemens, Jonas S. Erjefält, Caroline Sandén, Hans Jürgen Hoffmann, Marc Kästle, Ramona Schmid, Kevin Menden, Denis Delic

**Affiliations:** 1 Immunology and Respiratory Disease Research, Boehringer Ingelheim Pharma GmbH & Co. KG, Birkendorfer Straße 65, 88397 Biberach-an-der-Riss, Germany; 2 Boehringer Ingelheim Ltd, Burlington, Canada; 3 Medetect AB, Lund, Sweden; 4 Lund University, Lund, Sweden; 5 University of Aarhus, Aarhus, Denmark

**Keywords:** RORgt, IL-17, IL-22, Severe asthma, Inhibitor, Mouse model, Gene signature

## Abstract

**Background:**

RORγt is a transcription factor that enables elaboration of Th17-associated cytokines (including IL-17 and IL-22) and is proposed as a pharmacological target for severe asthma.

**Methods:**

IL-17 immunohistochemistry was performed in severe asthma bronchial biopsies (specificity confirmed with in situ hybridization). Primary human small airway epithelial cells in air liquid interface and primary bronchial smooth muscle cells were stimulated with recombinant human IL-17 and/or IL-22 and pro-inflammatory cytokines measured. Balb/c mice were challenged intratracheally with IL-17 and/or IL-22 and airway hyperreactivity, pro-inflammatory cytokines and airway neutrophilia measured. Balb/c mice were sensitized intraperitoneally and challenged intratracheally with house dust mite extract and the effect of either a RORγt inhibitor (BIX119) or an anti-IL-11 antibody assessed on airway hyperreactivity, pro-inflammatory cytokines and airway neutrophilia measured.

**Results:**

We confirmed in severe asthma bronchial biopsies both the presence of IL-17-positive lymphocytes and that an IL-17 transcriptome profile in a severe asthma patient sub-population. Both IL-17 and IL-22 stimulated the release of pro-inflammatory cytokine and chemokine release from primary human lung cells and in mice. Furthermore, IL-22 in combination with IL-17, but neither alone, elicits airway hyperresponsiveness (AHR) in naïve mice. A RORγt inhibitor specifically blocked both IL-17 and IL-22, AHR and neutrophilia in a mouse house dust mite model unlike other registered or advanced pipeline modes of action. Full efficacy versus these parameters was associated with 90% inhibition of IL-17 and 50% inhibition of IL-22. In contrast, anti-IL-17 also blocked IL-17, but not IL-22, AHR or neutrophilia. Moreover, the deregulated genes in the lungs from these mice correlated well with deregulated genes from severe asthma biopsies suggesting that this model recapitulates significant severe asthma-relevant biology. Furthermore, these genes were reversed upon RORγt inhibition in the HDM model. Cell deconvolution suggested that the responsible cells were corticosteroid insensitive γδ-T-cells.

**Conclusion:**

These data strongly suggest that both IL-17 and IL-22 are required for Th2-low endotype associated biology and that a RORγt inhibitor may provide improved clinical benefit in a severe asthma sub-population of patients by blocking both IL-17 and IL-22 biology compared with blocking IL-17 alone.

**Supplementary Information:**

The online version contains supplementary material available at 10.1186/s12931-021-01743-7.

## Background

Mild-to-moderate asthma is generally well controlled with inhaled corticosteroids and long acting β_2_ agonists, but a severe, treatment-refractory population remains. Approximately half of this severe population exhibits a phenotype driven largely by Th2-mediated inflammation which is partly controlled by oral corticosteroids and is expected to be further served by emerging biologics (anti-IL-4R, anti-IL-13, anti-IL-5, anti-IgE) and small molecules (e.g. CRTH2). However, the remaining severe asthmatic population comprises a more heterogeneous mixture of Th2-low endotypes, in which the anticipated unmet medical need is high [1]. Emerging data from the ongoing North American Severe Asthma Research Program (SARP) [2], European Unbiased Biomarkers for the Prediction of Respiratory Disease Outcomes (U-BIOPRED) [3] and Refractory Asthma Stratification Programme (RASP-UK) [4] studies may help to further characterise and deconvolute these Th2-low endotypes.

RAR-related orphan receptor gamma t (RORγt) is a predominantly lymphocyte restricted transcription factor that drives a Th17-like phenotype with the elaboration of associated cytokines such as IL-17A, IL-17F, IL-22, IL-26 and the expression of IL-23 receptor [5].

The Th17 pathway has been touted as a therapeutic target for severe Th2-low asthma [6]. Serum IL-17 has been reported to be elevated in patients with severe asthma [7, 8] and IL-17-positive cells are found in bronchial biopsies from severe asthmatics [9]. The Th17 pathway has been linked with refractory therapeutic responses to corticosteroids; severe asthmatic patients whose peripheral lymphocytes release more IL-17 exhibit poorer lung function responses to oral corticosteroids [10] and increased pulmonary IL-22 has been reported in steroid refractory asthma [11] and furthermore, serum IL-26 has also been reported to be elevated in uncontrolled asthma patients [12]. Mechanistic support for the link between Th17 and Th2-low asthma has been supplied from adoptive Th17 cell transfer experiments in mice that result in pulmonary neutrophilia and airway hyper-responsiveness (AHR) [13, 14]. Despite these links between the Th17 pathway and severe Th2-low asthma, in a large double-blind, placebo-controlled phase 2 study, the anti-IL-17 RA antibody, brodalumab, overall did not meaningfully impact quality of life or lung function parameters in inadequately controlled moderate to severe asthma [15], although a post hoc analysis suggested benefit in a sub-population of patients with high bronchodilator reversibility. Furthermore the anti-IL-17A neutralizing antibody secukinumab failed to improve symptom scores in a small severe asthma study [16]. With a lack of clinical success with anti-IL-17 approaches, a broader more upstream approach could be considered.

The aim of this study was to assess whether a RORγt inhibitor has potential utility in the treatment of a sub-set of severe asthma patients using a combination of analysis of severe asthma lung tissue and effects of a RORγt inhibitor in cellular and animal models that recapitulate elements of severe asthma relevant biology.

## Materials and methods

### Primary human bronchial smooth muscle cells and small airway epithelial cells in air–liquid interface culture

Primary human bronchial smooth muscle cells and small airway epithelial cells (both Lonza, passages 4 and 3, respectively) were cultured and differentiated according to the manufacturer’s instructions. Cells were stimulated with IL-17A, IL-22 or IL-26 and cytokine concentrations were measured using an MSD UPLEX pro-inflammatory 10-spot kit (Meso Scale Discovery) according to manufacturers’ instructions.

### Mouse models

A novel cytokine lung instillation was developed. Female Balb/c mice; 8–10 weeks (Charles River) were transiently anaesthetized with 3–4% Isoflurane, fixed in a supine position, the upper thorax illuminated, and the jaws opened, the tongue gently displaced and an external light source used to illuminate the vocal chords. 50 µL of either saline or either murine IL-17A, IL-22 or a combination of IL-17A and IL-22 instilled intratracheally (0.02 mg/ml of each in saline: final dose 1 µg of each cytokine). Within 24 h, lung function measurements performed (Scireq flexiVent); Mice were anaesthetized by intraperitoneal injection of Narcoren® (Narcorene i.p. 6 mg/ml, Rompun 0.25 mg/ml, administration volume 10 ml/kg). An intravenous cannula was inserted in the tail vein and fixed. After tracheostomy a tracheal cannula was inserted and fixed by a ligature. The tracheal cannula was connected to an integrated ventilation and lung function measurement device (Scireq flexiVent, Emka Technologies, Paris, France) with the software version 7.2. The spontaneous respiration was suppressed by intravenous administration of pancuronium bromide (0.8 mg/kg at a volume of 5 ml/kg). Ventilation was conducted with a tidal volume of 6.5 ml/kg, a breathing frequency of 150 breaths/ minute and a positive end expiratory pressure of 3 cmH2O using the template “FlexiVent FC-Mouse Default-rel B”. A “SnapShot-150 v7.0 (baseline airway resistance and compliance) and PVs-P v7.0” (pressure–volume loop; PVs-P) measurement was conducted. A dose response curve of Acetylcholine (Ach) was subsequently constructed: Ach was cumulatively administered in 4 different doses (50-;100-; 200-; 300 µg/ml, 50 µl i.v.) into the blood vessel of the tail. Before each Ach-administration, a SnapShot-150 was conducted. After administration of Acetylcholine, 8 further SnapShot-150 s were conducted over 2–3 min. Finally, the next Ach dose was given and the measurements repeated. After lung function measurement animals were euthanized by intravenous pentobarbital injection (80 mg/kg Narcoren®) and the lungs lavaged. Differential cell counts in BAL samples were performed in a Sysmex XT-1800i using a customised gating strategy. The gating strategy has been previously validated in three separate experiments against manually differentiated cell counts from cytospins. The cytokine KC concentrations measured by ELISA (R&D Systems).

A previously described Mouse house dust mite (HDM) model [17] was also studied. On day 0, Balb/c mice received a sub-cutaneous injection of 100 µg HDM extract (D. pteronyssinus; Greer Laboratories) in 100 µl of Freund´s Complete Adjuvant (Difco, BD Biosciences). On days 14 and 15, 50 µL of either saline or 25 µg HDM was instilled intratracheally. BIX119 in 0.5% hydroxyethylcellulose / 0.01% Tween20 was dosed orally twice daily. On day 16, lung function and bronchioalveolar lavage was performed as above. The right lung was excised for NGS. The left lung was excised, and the cells dissociated using a gentleMACS Dissociator and the mouse lung dissociation kit (both Miltenyi Biotec GmBH). The tissue homogenate was strained, and the single cell suspension washed and counted. T-cells were activated with ionomycin (250 ng/mL) and phorbol myristate acetate (10 ng/mL) in the presence of monensin (sodium ionophore to prevent the extracellular release of cytokines) for 4 h at 37 °C. Cells were washed and stained for CD3 (FITC rat anti-mouse CD3 molecular complex; BD Pharmingen) and CD4 (APC-H7 Mouse Anti-mouse CD4; BD Pharmingen), before fixation and permeabilization with Transcription Factor Buffer Set (BD Pharmingen). Cells were subsequently stained for T-bet (BV421-mouse anti-T-bet), GATA3 (BV421 mouse anti-GATA3), RORγt (BV421 mouse anti-RORγt), IFNγ (AF 647 rat anti-mouse IFNγ), IL-4 (AF 647 rat anti-mouse IL-4) and IL-17A (AF 647 rat anti-mouse IL-17A; all BD Pharmingen). FSC versus SSC plots were generated using an LSR II flow cytometer (BD Pharmingen) to identify single intact cells which were gated into a CD3 versus CD4 plot to identify CD3 + /CD4 + double-positive cells which were then gated into an IL-17 versus RORγt plot to identify IL-17 + /RORγt + double-positive cells.

### toRNA-Seq data generation and data processing

Mouse lung and human precision cut lung slices (PCLS) were homogenised in a Precellys-24 system (Bertin Corp.), RNA isolated using RNACleanXP beads and amplified using TruSeq Stranded Total RNA Library Prep Kit (Illumina). Fragments were assessed using a Fragment Analyzer Standard Sensitivity RNA Analysis Kit (AATI). Sequenced reads were mapped against reference genome GRCm38.p4 using the STAR mapping tool version 2.5.2a [17]. Raw read counts were calculated using Subread [18]. Differential expression analysis was performed with limma [19] and edgeR [20].

### Immunohistochemistry in severe asthma bronchial biospies

Immunohistochemistry (IHC) for IL-17 was performed in bronchial biopsies and antibody specificity was further confirmed via in situ hybridization (ISH). IL-17 mRNA was visualized using the RNAscope 2.5 FFPE assay kit (Advanced Cell Diagnostics). IL-17 mRNA ISH was combined with IL-17 IHC (R&D; AF-317-NA; 1:300) and additionally combined with IHC detection of MPO (Dako; A0398; 1:10,000), CD3 (Dako, M725401-2; 1:1,000), tryptase (Millipore; CHE MAB1222A; 1:12,000)) and CD68 (Dako; M0814; 1:3,000), to detect neutrophils, T lymphocytes, mast cells, or macrophages. Appropriate isotype controls were included (rabbit IgG, Dako, X093602-2; mouse IgG1, Dako, X093191-2; and goat serum, Dako, X0907). Detection was achieved with the appropriate secondary antibodies (rabbit anti-goat IgG-HRP, Dako, P0449 and Envision™ FLEX/HRP to detect rabbit and goat IgG, Dako, K8010). The IHC procedure was automated using an automated immunohistochemistry robot (Autostainer Plus, Dako). Briefly, sections were pretreated through HIER, blocked with endogenous enzyme block (EnVision™ FLEX Peroxidase-Blocking Reagent, Dako) for 10 min followed by incubation with serum free protein block (Dako) for 10 min before the incubation with the primary anti-MPO and anti-tryptase antibodies. After a washing step the primary antibodies were detected by secondary antibodies conjugated to HR-Peroxidase (HRP) and DAB (brown chromogen). This was followed by incubation with anti-IL-17, which was then detected by sequential incubation with rabbit anti-goat-HRP, goat anti rabbit/mouse-HRP (EnVision™ FLEX/HRP) and the chromogen Vina green. Hematoxylin was used as background staining (blue nuclei), the tissue was dehydrated and the slides were then mounted under glass coverslips with Pertex mounting medium. Each section was digitalized, the numbers of IL-17 positive cells counted manually in each biopsy and the total area of analyzed tissue was measured by image analysis (CCV, Medetect in house image analysis software).

### Bioinformatic data analysis

Unbiased cluster analysis was performed using transcriptome signatures [21, 22] containing probesets which yielded reliable signals above noise level. Expression levels were mean centered and averaged over the Th17 signature genes to generate a Th17 score. Samples were then assigned to patient clusters according to these scores (cut-off: 0), as described previously [23].

For the cross-study correlation analysis, data was leveraged from the published UBIOPRED [24] and SARP [25] cohorts. A differential expression was quantified by log-ratios and p-values. Each data set was filtered with a cut-off of p.adj.fdr <  = 0.05. Pearson correlation was calculated between log-ratios of each pair of studies. The lower left part of Fig. 5a shows the log ratios of the respective studies with the coloring representing the density of the data points. The upper right part of the figure indicates the number of data points (“n”), p-value and r of the correlation with the color of the square representing r. All computational analyses were performed in R [26].

For cell type quantification, the digital cell quantification (DCQ) algorithm developed by Altboum et al. [27] was applied as implemented in the ComICS R package 24 [28] using the dcq function with default parameters (number of repeats = 3, lambda min = 0.2). The DCQ algorithm uses elastic net [29] to model the change in gene expression. Cell type quantification is based on an immune cell compendium of 213 immune cells based on published data [30, 31]. As input, counts per million expression values were used. The resulting table with the average cell type quantity values were averaged for every group and subsequently filtered for cell types with average quantity values below 2.0. All heatmaps were plotted using the pheatmap package.

### Cell deconvolution

Genes were ranked according to their fold change and cell type specific gene lists [32] were used to calculate overlaps between every window of size 200 in the fold change ranked gene list and every cell type gene list. Overlap with highly specific genes in the 2000 genes long ranking were weighted stronger than genes that fall behind in the ranking using a “sliding window” approach. Overlap with the top 100 genes was multiplied with 20, with the next 100 genes with 19, going forth until 1 for the last 100 genes.

### GSVA analysis

Gene set variation analysis was performed using the GSVA R package [33] and published Transcriptomic Associated Cluster genesets [34]. Genes were substituted with mouse orthologs and heatmaps generated using the pheatmap R package [35].

### Statistical analyses

Data were present as either individual values or as mean ± S.E.M. Statistical differences were compared with the one-way variance analysis (ANOVA) followed by the Dunnett’s test to correct for multiple comparisons. The limit of the significance was taken as p values less than 0.05 (p < 0.05). The percentage inhibition was calculated from the mean value in the positive group according to: % inhibition = 100-(Y/K1)*100. With K1 being the mean value of the vehicle-treated negative control group subtracted from the mean value of the vehicle-treated positive control group and Y being the mean value of the vehicle-treated negative control group subtracted from the mean value of the respective compound-treated group. These tests were performed using GraphPad Prism version 6.01 for Windows, GraphPad Software, La Jolla, California, USA, “www.graphpad.com”.

## Results

### Identification of a Th17 patient endotype in severe asthma

**Fig. 1:**
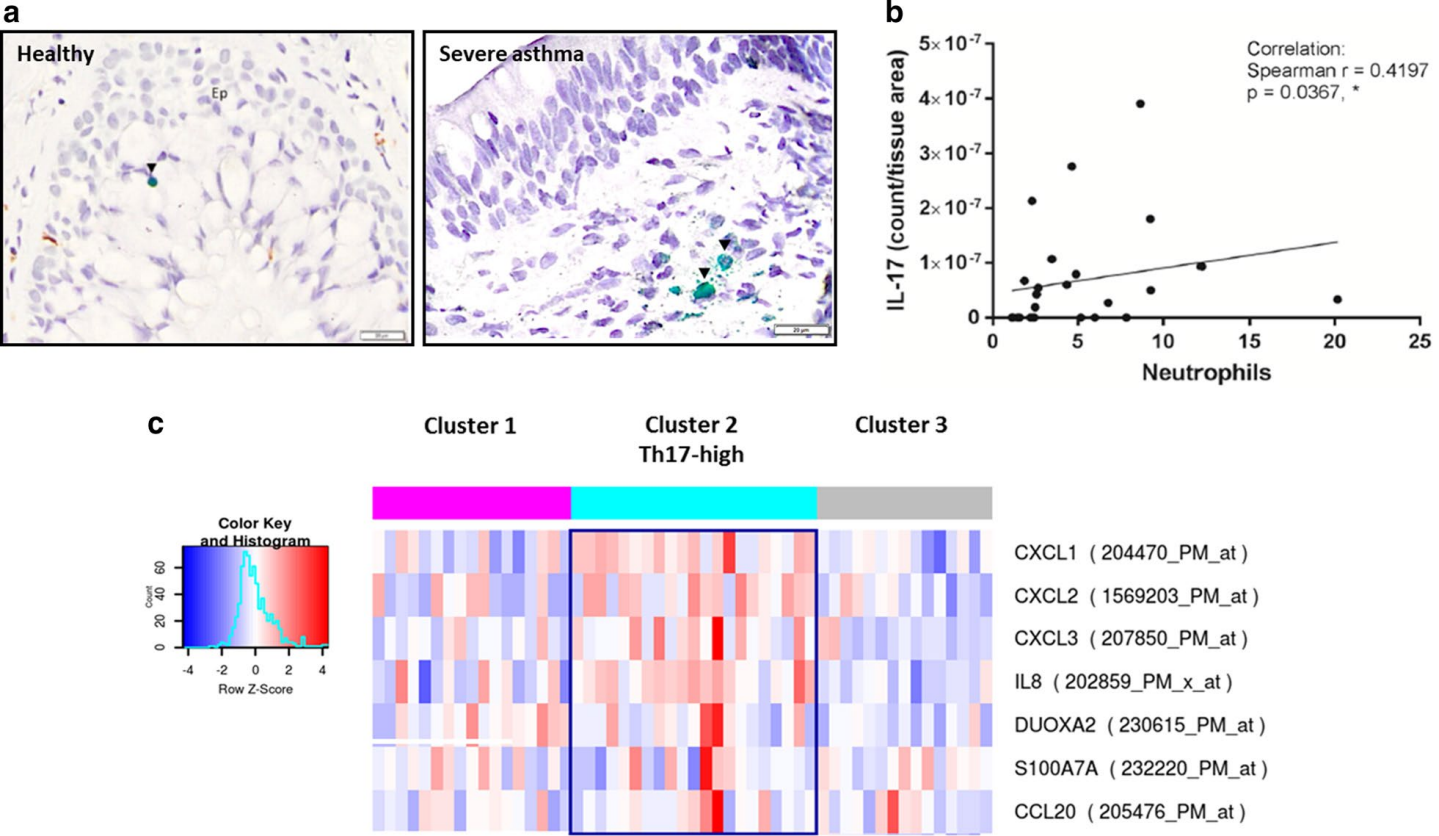
Representative photomicrographs of bronchial biopsies from a healthy individual and a severe asthma patient (**a**) stained for IL-17 (green staining; arrows). The bar indicates 20 µm. Correlation between the number of IL-17 positively staining cells and tissue neutrophils in severe asthma patients (**b**). Cluster analysis of UBIOPRED bronchial biopsy transcriptome using Th2 and Th17 surrogate genes published by Choy et al., SciTransMed, 2015 (**c**)

Bronchial biopsies from severe asthma patients and healthy controls (Table 1) were stained for IL-17 by immunohistochemistry (IHC). Commercial anti-IL-17 antibodies have recently been shown to non-specifically stain neutrophil and mast cell granules. Consequently, a double staining IHC protocol was developed to first conceal the confounding neutrophil and mast cell immunoreactivity followed by detection of true IL-17 positive cells. Staining specificity was further confirmed by co-staining for CD3 immunoreactivity and IL-17 mRNA by in situ hybridization (data not shown). Overall, the numbers of IL-17-positive cells in each bronchial biopsy was low and only 5 IL-17 positive cells were identified from 13 healthy biopsies and 11 IL-17 positive cells identified from 25 severe asthma biopsies (representative photomicrograph from both a healthy individual and a severe asthma patient shown in Fig. 1a), although 15 out of 25 severe asthma bronchial biopsies (60%) contained IL-17 positive cells compared to 6 out of 13 biopsies from healthy volunteers (46%). There was a weak, but statistically significant correlation between number of IL-17-positive cells and number of neutrophils (both normalized for tissue area quantified) in the severe asthma biopsies (Fig. 1b). Finally, the published Th17 transcriptome signatures (CXCL1, CXCL2, CXCL3 and IL-8) [21] and (DUOXA2, S100A7A and CCL20) [22] were applied to the transcriptome from an additional larger published cohort of severe asthma bronchial biopsies from 107 patients (U-BIOPRED) and an unbiased cluster analysis identified 3 clusters, one of which is a distinct Th17-high cluster present in 21 out of the 53 biopsies (40%) (Fig. 1c).

**Table 1:** Characteristics of asthma patients and healthy control subjects

	Healthy controls	Severe asthma
N	8	25
Gender (M/F)	3/5	10/15
Age (years)	23 (21–39)	51 (17–66)
Inhaled CS (yes/no/unknown)	0/8/0	25/0/0
Oral CS (yes/no/unknown)	0/8/0	25/0/0
LABA (yes/no/unknown)	0/8/0	24/1/0
SABA (yes/no/unknown)	0/8/0	21/4/0
FEV1% predicted	98.1 (72.1–116.4)	65.6 (32.4–93.8)
Smokers (yes/ex/never/unknown)	0/0/8/0	1/12/8/4
Number of biopsies	13	25
Number of biopsies with IL-17 positive cells (%)	6 (46%)	15 (60%)

A single biopsy was taken from 25 severe asthma patients (N = 25 total) whereas a single biopsy was taken from 3 healthy volunteers and a double biopsy taken from a further 5 healthy volunteers (N = 13 total)

*CS* corticosteroids, *LABA* long acting beta-agonist, *SABA* short acting beta-agonist, *FEV*_*1*_ forced expiratory volume in 1 min

### Consequences of Il-17 and IL-22 pathway activation in vitro and in vivo

**Fig. 2:**
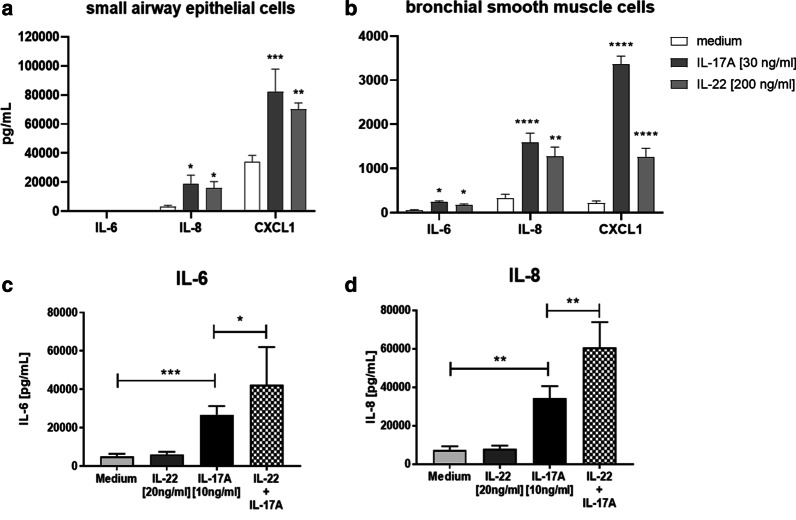
Human primary small airway epithelial cells differentiated in air–liquid interface culture were stimulated for 24 h with 30 ng/ml IL-17 or 200 ng/ml IL-22 and the release of IL-6, IL-8 and CXCL1 was measured (**a)**. Primary human bronchial smooth muscle cells were stimulated for 24 h with 30 ng/ml IL-17 or 200 ng/ml IL-22 and the release of IL-6, IL-8 and CXCL1 was measured (**b**) or stimulated for 24 h with 10 ng/ml IL-17 + 20 ng/ml IL-22 and the release of IL-6 (**c**) and IL-8 (**d**) was measured. Data are expressed as mean ± SEM of three independent experiments. Statistical analysis was performed using a non-parametric Kruskal–Wallis test with Dunn’s multiple comparison (*P < 0.05, **P < 0.01, ***P < 0.005, ****P < 0.001)

**Fig. 3:**
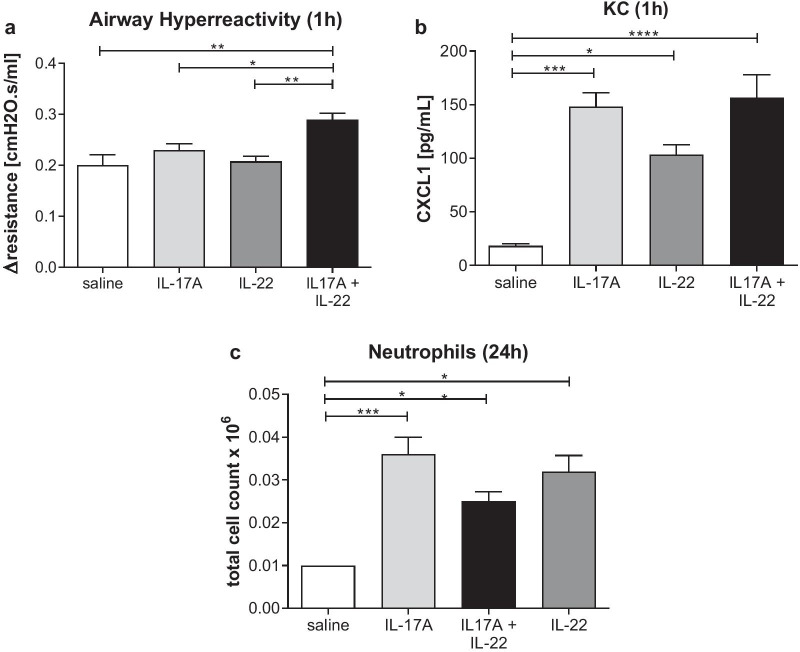
Female Balb/c mice were challenged intratracheally with saline (N = 4) IL-17A alone (N = 8), IL-22 (N = 8) alone or with the combination (N = 8). After 1 h, airway hyperreactivity (**a**) and lung tissue CXCL1 (**b**) were measured. After 24 h, bronchioalveolar lavage fluid neutrophil count (**c**) was measured. Data are expressed as mean ± SEM. Statistical analysis was performed using a non-parametric Kruskal–Wallis test with Dunn’s multiple comparison (*P < 0.05, **P < 0.01, ***P < 0.005, ****P < 0.001)

To assess functionality and interplay between Th17-associated cytokines in the lung, human primary small airway epithelial cells differentiated in air–liquid interface culture were initially stimulated with IL-17A or IL-22 for 24 h and significant increases in IL-8 and CXCL1 (Fig. 2a) were observed. IL-17A was selected because IL-17A, but not IL-17F, was detectable in the mouse HDM model. Primary human bronchial smooth muscle cells were also stimulated with IL-17A or IL-22 for 24 h and increases in IL-6, IL-8 and CXCL1 (Fig. 2b) were observed. Because the biological response was smaller in the smooth muscle cells compared with the epithelial cells, the smooth muscle cells were selected for further experiments in which sub-maximal efficacious concentrations of IL-17A and IL-22 resulted in an over-additive release of IL-6 (Fig. 2c) and IL-8 (Fig. 2d) from the smooth muscle cells. Intratracheal instillation of a combination of IL-17A and IL-22 into mouse lungs increased acetylcholine-induced AHR, but not either IL-22 alone or IL-17A alone (Fig. 3a). In contrast, instillation of IL-17A alone, IL-22 alone or a combination increased lung concentration of KC (CXCL1; Fig. 3b) and induced airway neutrophilia (Fig. 3c) that were not augmented by the addition of IL-22.

### Functional inhibition of Th17 pathway in vivo

**Fig. 4:**
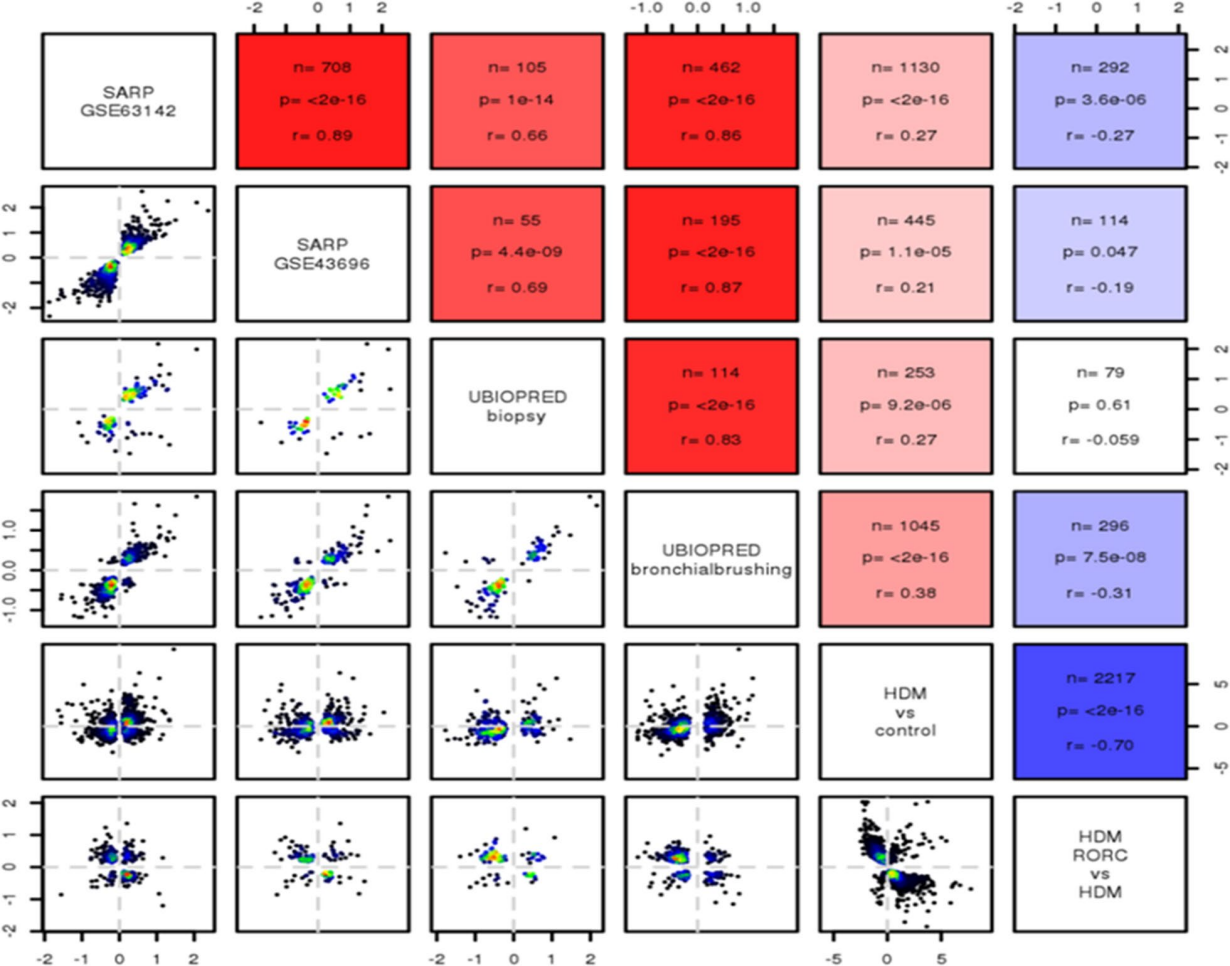
Correlation of DEGs (log ratios, filtered by p.adj.fdr < 0.05) in SARP and UBIOPRED severe asthma samples vs. healthy controls against lung DEGs in the mouse HDM model ± RORγt inhibitor. Red shading indicates a positive correlation (r > 0) and blue shading indicates a negative correlation (r < 0). P values and R values are provided

**Fig. 5:**
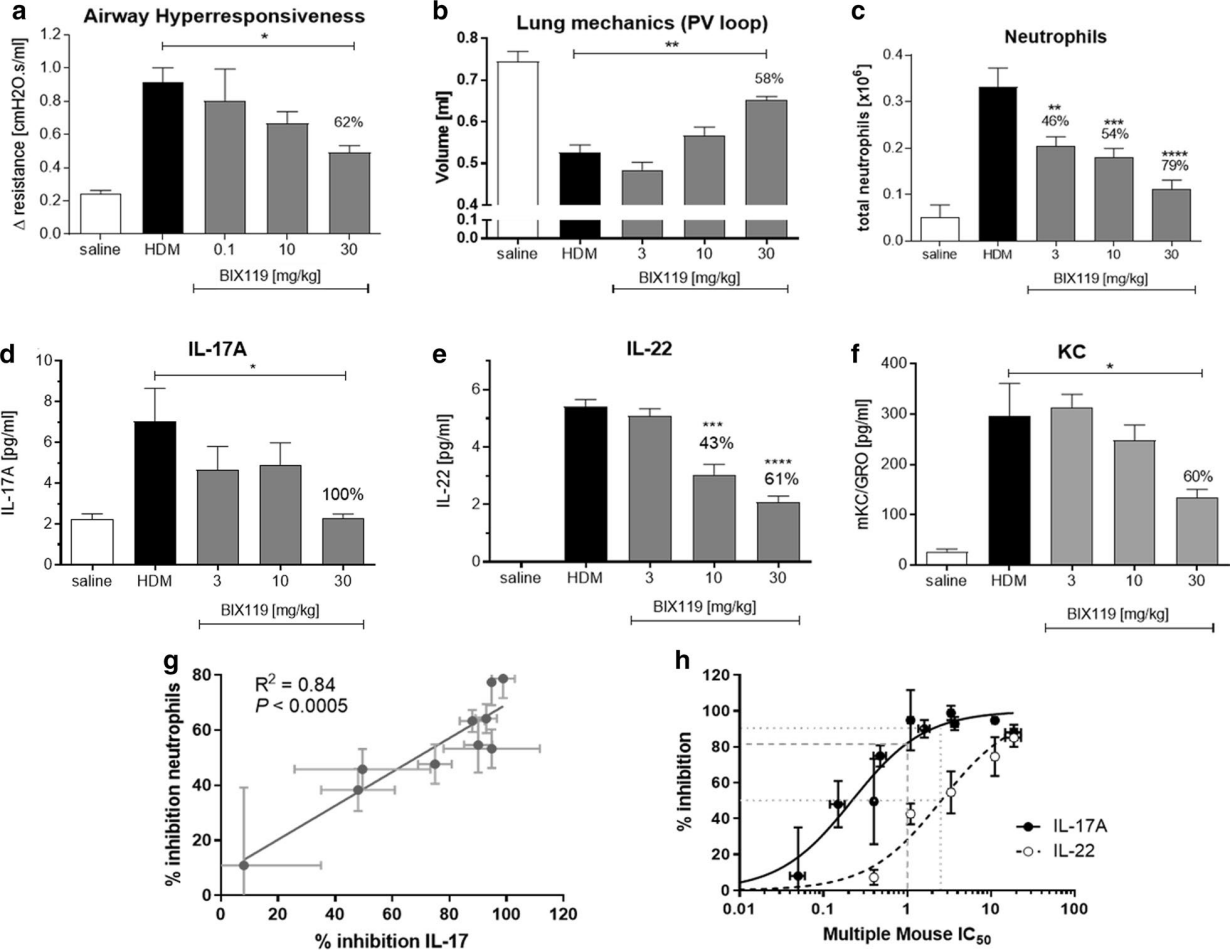
Female Balb/c mice were sensitized with house dust mite (HDM) in complete Freunds adjuvant intraperitoneally on days 0 and 1 and subsequently challenged intratracheally with saline (N = 6) or HDM in saline (N = 8) on days 14 and 15. BIX119 was dosed twice daily on days 14 and 15 by oral gavage. Mice were sacrificed on day 16 and airway hyperresponsiveness to acetylcholine (**a**), pressure–volume loops (**b**), and bronchioalveolar lavage fluid neutrophils (**c**), IL-17 (**d**), IL-22 (**e**) and KC (**f**) were measured. The relationship between bronchioalveolar lavage fluid neutrophil count (plotted as percentage inhibition from the HDM-alone treatment group) and lung tissue IL-17 (**g**) and lung tissue IL-17 / IL-22 (plotted as percentage inhibition from the HDM-alone treatment group) and trough plasma exposure (plotted as multiple of the mouse whole blood IL-17 IC_50_) (**h**) is shown. Data are expressed as mean ± SEM. Statistical analysis was performed using a non-parametric Kruskal–Wallis test with Dunn’s multiple comparison (*P < 0.05, **P < 0.01, ***P < 0.005, ****P < 0.001)

Next we sought to assess the impact of functional inhibition of the Th17 pathway using a novel, potent and selective RORγt inhibitor, BIX119 [36], in a mouse house dust mite (HDM) model of mixed pulmonary inflammation. This model was selected as a result of a positive correlation between deregulated genes in the lungs of these mice and from severe asthma lung biopsies from both UBIOPRED and SARP cohorts (Fig. 4; P values range between 1 × 10^–5^ and 2 × 10^–16^ depending on human dataset used for comparison) suggesting that this model recapitulates a significant number of severe asthma relevant pathways. BIX119 dose-dependently improved both HDM-induced, acetylcholine provoked AHR (Fig. 5a) and pulmonary mechanics as assessed by pressure–volume loops (Fig. 5b). This was associated with a reduction in airway neutrophils (Fig. 5c), and the cytokines IL-17 (Fig. 5d), IL-22 (Fig. 5e) and KC (Fig. 5f). Interestingly BIX119 had no effect on the Th2-related endpoints of airway eosinophils or cytokines IL-4 or IL-5 (Additional file 1: Figure S1). There was a linear relationship between the extent of neutrophil reduction and lung IL-17 inhibition (Fig. 5g) and neutrophil reduction and IL-22 inhibition (data not shown) suggesting a direct relationship between the two. Furthermore, there was a sigmoidal relationship between both IL-17 and IL-22 inhibition in the lung and trough plasma exposure of BIX119 (Fig. 5h). Interestingly, the exposure–response curve for IL-17 was leftward shifted compared to IL-22, but at trough plasma exposures that result in 90% inhibition of IL-17, IL-22 was still inhibited by 50% (Fig. 5h, dotted grey line). Furthermore, achieving the cellular IC_50_ in whole blood (120 nM) at trough delivers 80% inhibition of lung IL-17 (Fig. 5h, dashed grey line).

**Fig. 6:**
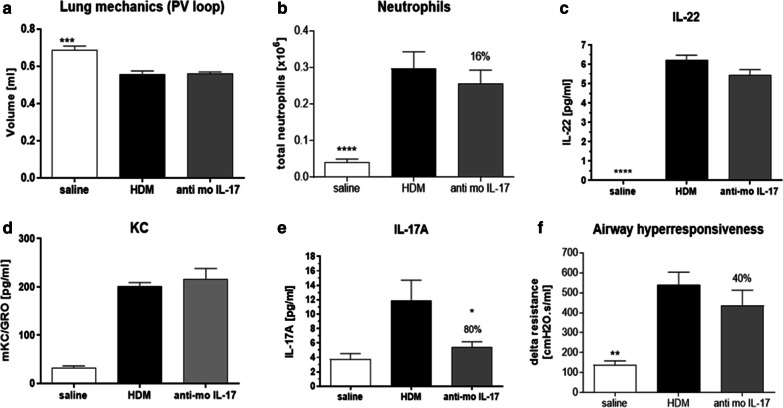
Female Balb/c mice were senitised with house dust mite (HDM) in complete Freunds adjuvant intraperitoneally on days 0 and 1 and subsequently challenged intratracheally with saline (N = 6) or HDM in saline (N = 8) on days 14 and 15. Anti-mouse IL-17 blocking antibody or isotype control (400 µg) was dosed intraperitoneally on day 13. Mice were sacrificed on day 16 and pressure–volume loops (**a**), and bronchioalveolar lavage fluid neutrophils (**b**), IL-22 (**c**), KC (**d**) and IL-17 (**e**) and airway hyperresponsiveness to acetylcholine (**f**) were measured. Data are expressed as mean ± SEM. Statistical analysis was performed using a non-parametric Kruskal–Wallis test with Dunn’s multiple comparison (*P < 0.05)

In contrast, in the same mouse model, an IL-17 blocking antibody did not improve pulmonary mechanics (Fig. 6a), pulmonary neutrophils (Fig. 6b) or levels of IL-22 (Fig. 6c) or KC (Fig. 6d), despite reducing IL-17 levels by 80% /Fig. 6e). A modest 40% improvement in airway hyperreactivity (Fig. 6f) was observed. In this mouse model, the reduction of non-Th2 relevant endpoints (pulmonary neutrophilia and IL-8) and Th17 relevant endpoints (IL-17) observed with BIX119 was not achieved to the same extent by other launched and pipeline treatments for severe asthma (anti-IgE, anti-IL-5, anti-IL-4R, anti-TSLP, anti-IL-33 antibodies, monteleukast or oral corticosteroids; Table 2).

**Table 2:** Efficacy profiles of RORγt inhibition versus launched and pipeline mechanisms of action in the mouse house dust mite model

Endpoint		RORγt	Anti-IL-5	Anti-IL4 / anti-IL-13	Anti-IgE	CysLT1	oCS	Anti-TSLP	Anti-IL-33
Phenotypic	Airway hyperreactivity	58%*	0%	0%	0%	15%	65%*	62%*	n/d
	Pulmonary mechanics	62%^#^	6%	20%	29%	0%	22%	0%	n/d
Th2	Eosinophils	0%	78%	8%	43%*	10%	21%	53%^#^	0%
	IL-4	0%	50%^#^	49%*	92%^#^	45%*	31%	0%	0%
	IL-5	0%	100%*	40%*	0%	0%	17%	46%*	30%
non-Th2	Neutrophils	79%^§^	40%*	3%	0%	0%	59%^#^	9%	0%
	Macrophages	0%	55%^#^	17%	0%	0%	97%^§^	13%	0%
	IL-1β	0%	21%	26%	0%	8%	56%^#^	10%	0%
	IL-6	0%	60%*	50%*	0%	22%	43%	0%	0%
	IL-8	60%*	0%	25%	0%	14%	20%	0%	0%
	IL-17	90%^§^	0%	0%	0%	0%	67%^#^	0%	0%
	IFN-γ	0%	44%	58%	0%	0%	45%	0%	0%

The effect size of BIX119 in the HDM mouse model (RORγt) versus lung function (phenotypic endpoint), Th2 and non-Th2-associated endpoints was compared with other clinical relevant modes of action anti-IL-5 (rat-anti-mouse IL-5; TRFK-5; 300 µg i.p.), anti-IL4 + anti-IL-13 (as a surrogate for anti-IL-4R; rat anti-mouse IL-4; MAB; 300 µg i.p. and rat anti-mouse IL-13; MAB413; 300 µg i.p.), anti-IgE (rat anti-mouse IgE; antibody1-5; 300 µg i.p.), CysLT1 (monteleukast; 25 mg/kg/day p.o.), oral corticosteroids (oCS; prednisolone; 4 mg/kg/day p.o.), anti-TSLP (rat anti-mouse TSLP; MAB555; 300 µg i.p.) and anti-IL-33R (rat anti-mouse IL-33R; MAB10041; 250 µg i.p.) in the same model. All treatments were administered on day 13, 24 h prior to challenge. Antibodies were dosed just once on day 13, small molecule inhibitors were dosed on days 13, 14 and 15. n/d not determined, *P < 0.05, ^#^P < 0.01, ^§^P < 0.005

**Fig. 7:**
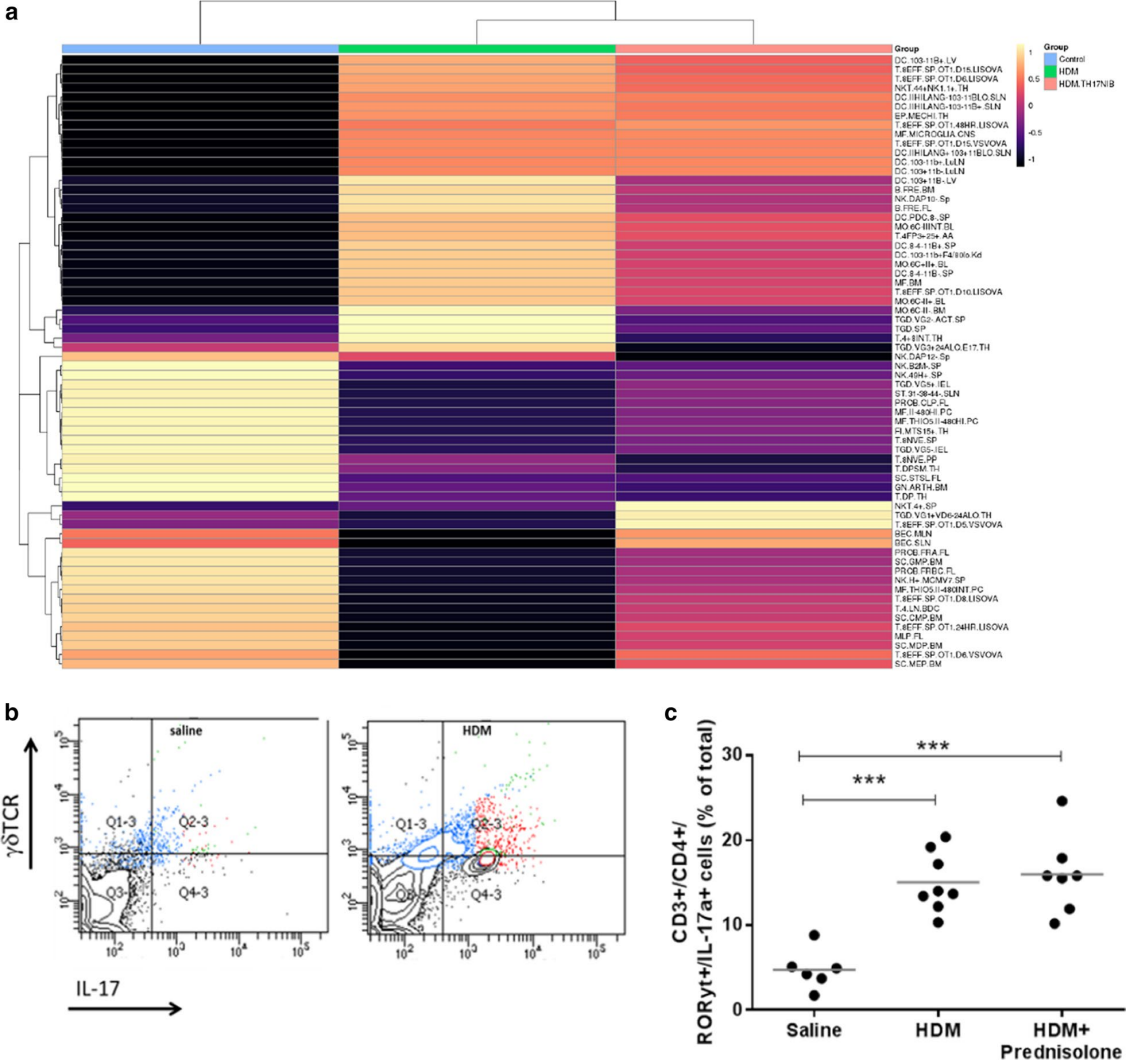
Female Balb/c mice were senitised with house dust mite (HDM) in complete Freunds adjuvant intraperitoneally on days 0 and 1 and subsequently challenged intracheally with saline (N = 6) or HDM in saline (N = 8) on days 14 and 15. **a** Next generation sequencing was performed on lung tissue and cell deconvolution performed using a DCQ algorithm. Heatmap of z-score transformed scores calculated by the digital cell quantication algorithm for all three different groups. High values correspond to predicted enrichment of the cell type indicated in the rows. Only cells which have a significant change in quantity between conditions are shown. Highest up-regulation (light orange) to highest down-regulation (dark purple). **b** Lung digestion and flow cytometry was performed on the single cell suspension. **c** Effect of oral prednisolone 10 mg/kg b.i.d. on the presence of γδ-T-cells in the lungs of HDM treated mice. Data are expressed as mean ± SEM. Statistical analysis was performed using a non-parametric Kruskal–Wallis test with Dunn’s multiple comparison (***P < 0.005)

Next generation sequencing (NGS) of lung tissue mRNA coupled with cell deconvolution analysis identified genes associated with IL-17 and IL-22 expressing γδT-cells (Fig. 7a; black arrows) which was reversed upon treatment with BIX119. The presence of this predominantly IL-17 expressing lymphocyte was confirmed by flow cytometry analysis of a single cell lung digest (Fig. 7b). The presence of γδT-cells in this model was not reduced by oral corticosteroids (Fig. 7c). Interestingly, cell deconvolution showed only a small difference in neutrophil gene signatures between HDM-treated mice and controls (Fig. 7a) which is apparently in contrast to the larger increase in cell numbers observed (Fig. 5c). However, the neutrophil signature used for the deconvolution analysis is derived from resting peripheral neutrophils, whereas those neutrophils observed in the lung have been exposed to numerous activating factors which are likely to change the mRNA signature [37] which may explain this apparent discrepancy. NGS analysis also showed increases in CXCL1, CCL20 and IL-6 transcripts in HDM mouse lung which were reversed by treatment with BIX119 (data not shown). Increases in the same genes were also observed in bronchial biopsies from the U-BIOPRED consortium (GSE76225; data not shown).

### Mapping RORγt sensitive genes onto the Th17 severe asthma patient endotype

**Fig. 8:**
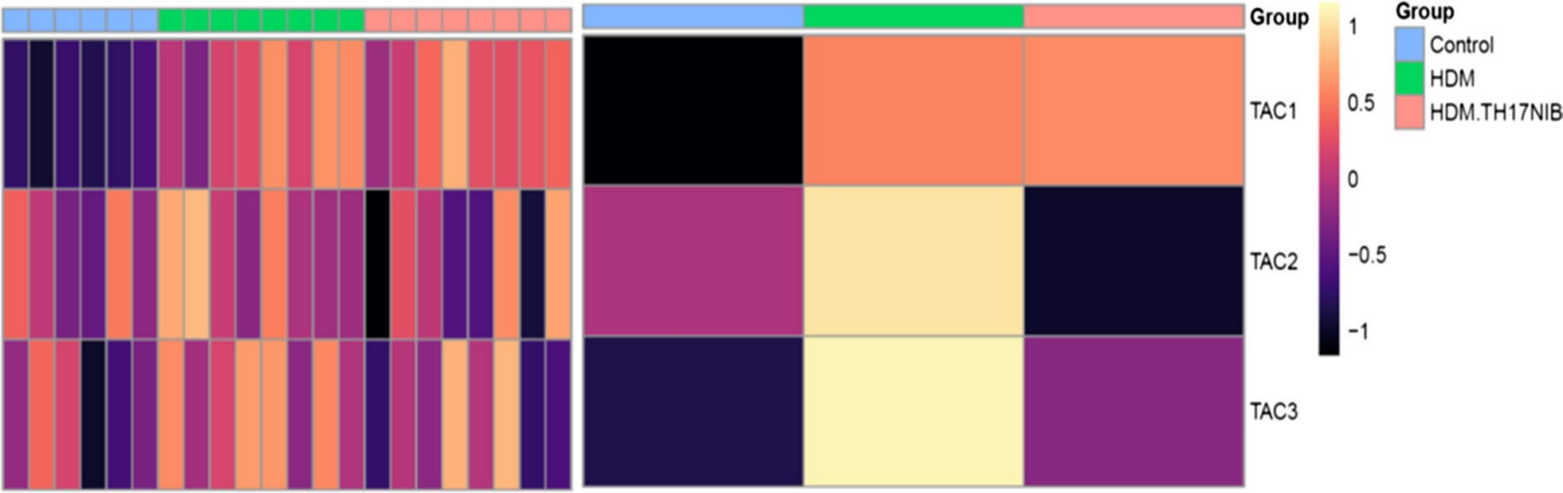
Lung DEGs from the HDM model ± RORγt inhibitor were compared with sputum DEGs from UBIOPRED that have been segregated according to the Transcriptomic-associated clusters (TACs) defined by Kuo et al. [38]. The left-hand side depicts DEGs in individual animals and the right-hand side depicts an averaged response within the treatment group. A purple color indicates no response, with lighter colors indicating an upregulation and darker colors indicating a downregulation

Differentially expressed genes (DEGs) in severe asthma endobronchial brushings (GSE63142, SARP), bronchial epithelial cells (GSE43696, SARP), epithelial brushings (GSE76226, U-BIOPRED) and bronchial biopsies (GSE76225, U-BIOPRED) were compared and a strong, positive correlation was observed between the datasets (r values between 0.66 and 0.89; P values between 4e−09 to 2e−16; Fig. 8a). Lung tissue DEGs in the mouse HDM model, compared to control animals, correlated positively with the clinical datasets, but less strongly than the correlations between the clinical datasets (r values between 0.21 and 0.38; P values between 1e−0 to 2e−16; Fig. 8a). Lung tissue DEGs in the HDM animals treated with BIX119 strongly and inversely correlated with DEGs in the HDM-only animals (r = − 0.70; P = 2e−16; Fig. 8a). Furthermore, lung tissue DEGs following treatment with BIX119 in the HDM model significantly and inversely correlated with DEGs in the SARP (r values between − 0.19 and − 0.27; P values between 0.047 to 3.6e−06; Fig. 8a) and U-BIOPRED bronchial brushing (r = − 0.31; P = 7.5e−08; Fig. 8a) datasets.

Finally, Kuo et al [34] published 3 transcription-associated clusters (TACs) based on hierarchical clustering of DEGs found in sputum from the U-BIOPRED study. Lung tissue DEGs from HDM-treated mice positively, and most strongly correlated with DEGs found in TAC2, and additionally DEGS from HDM mice treated with BIX119 negatively and most strongly correlated with DEGs found in TAC2 (Fig. 8b).

## Discussion

The aim of this study was to determine whether a RORγt inhibitor may have utility in treating a sub-set of patients with severe asthma. We employed a novel approach whereby RORγt-sensitive DEGs were identified in a HDM-driven mouse model of mixed Th1, Th2 and Th17 pulmonary inflammation, validated in IL-17 and IL-22 stimulated primary human lung cells and correlated with transcriptome profiles from severe asthma patients in the U-BIOPRED and SARP cohorts. As expected from the mechanism of action, BIX119, a potent and selective inhibitor of RORγt, blocked γδT-cell-mediated Th17 responses in the mouse, but neither Th1 (IFNγ; data not shown) nor Th2 responses. This was associated with improvements in both AHR and pulmonary neutrophilia, although when individual Th17-associated cytokines were instilled into naïve mouse lungs, IL-17 was associated with neutrophil recruitment, whereas IL-22 was required in combination with IL-17 to drive AHR. Similar findings have also been reported with a RORγt inverse agonist [39] although the clinical significance was not explored. In this study we also demonstrate that these effects are linked to 80% pathway engagement and trough plasma concentrations that cover cellular IC_50_ indicating that these are effects are specific and on-target. In the same model, blockade of IL-17 alone did not improve pulmonary mechanics and measures of inflammation (with only a modest improvement in AHR) strongly suggesting that additional mediators beyond IL-17 are required to drive this phenotype. This is supported by the over additive pro-inflammatory effects of IL-17 and IL-22 observed in airway smooth muscle and epithelial cells together with evidence that IL-17 and IL-22 are both required to elicit AHR in naïve mice and that BIX119 inhibits both IL-17 and IL-22 in a mouse house dust mite mouse model associated with improvements in lung function and neutrophilia, but not Th2-associated endpoints such as eosinophils, IL-4 and Il-5. Moreover, other launched treatments for moderate-to-severe asthma such as anti-IgE (omalizumab), anti-IL-5 (mepolizumab) and anti-IL-4/anti-IL-13 neutralizing antibodies (IL-4R; dupilumab), Singulair (leukotriene B4 receptor antagonist) and oral corticosteroids and pipeline products such as anti-TSLP and anti-IL-33 neutralizing antibodies did not reduce pulmonary neutrophilia, IL-17 and IL-8 to the same extent as RORγt blockade suggesting that a RORγt inhibitor will provide a unique mode of treatment for patients.

Th17 inflammation has long been associated with severe neutrophilic asthma [6–11, 21, 40–42], but here we show for the first time that AHR may be induced by Th17-associated cytokines independently from pulmonary neutrophilia. This may explain the lack of clinical efficacy with anti-IL-17 [16] and IL-17 receptor [15] approaches in severe asthma and that perhaps a broader approach is required to block additional Th17-associated cytokines such IL-22 and IL-26. This observation is consistent with findings from clinical studies in which blockade of CXCL2 effectively reduced neutrophil numbers in severe asthmatic patients was not associated with reduction of exacerbations nor improvement in asthma control or lung function [43, 44], further suggesting that modulating pulmonary neutrophil numbers may be somewhat dissociated from achieving clinical control. The finding that AHR is dependent upon both IL17 and IL-22 in mice is not consistent with the reported induction of smooth muscle sensitivity to muscarinic agonists by IL-17 in human and mouse precision cut lung slides [45], but we were unable to replicate these findings (data not shown). Insensitivity to corticosteroid in severe asthma has also been associated with the Th17 pathway in both humans [10, 11] and mouse models [14] and we show here that the cell type responsible for Th17 responses in the mouse house dust mite model is also insensitive to corticosteroid treatment.

In addition to chemokines, the Th17-associated cytokines IL-17 and IL-22 also induced IL-6 and IL-8 expression and release from primary human pulmonary cells and lung slices. IL-6 has been reported to be associated with loss of central airway function in asthma [46] and a recent analysis from the SARP consortia reported that a sub-population of severe asthmatics with elevated serum IL-6 experienced poorer lung function and higher exacerbation rates [47]. Elevated airway levels of IL-8 and neutrophils have also been described in severe asthmatics requiring acute mechanical ventilation [48].

IL-17 and IL-22 mRNA was not detectable in U-BIOPRED bronchial biopsies by gene array, however, the Th17-associated transcriptome signature described by Choy et al. [21] was identified and confirmed their findings that a sub-population containing such a signature exists. This sub-population predominantly contained patients with sputum neutrophils > 60%. The lack of Th17 cytokine mRNA would suggest the presence of only small numbers of Th17 lymphocytes in bronchial tissue driving downstream biology. We confirmed the presence of small numbers of Th17-cells in lung biopsies from severe asthma patients defined by their treatment with oral corticosteroids using a specific and novel IHC strategy to block non-specific staining and confirming the specificity with CD3 co-staining and ISH. This is consistent with the earlier findings of Al-Ramli et al. [9] who employed a more traditional IHC approach. Interestingly, the numbers of Th17-positive lymphocytes correlated with tissue neutrophil numbers.

The selection of the mouse HDM model to test BIX119 was based upon the elicited Th17 response observed. However, it should be acknowledged that no one mouse model recapitulates all aspects of human disease and only models’ certain aspects (in this case the pulmonary Th17 response). Nevertheless, DEGs in the mouse HDM model correlated positively with DEGs from bronchial biopsies in both SARP and U-BIOPRED cohorts. However, in a novel translational approach, DEGs that were reversed by inhibition of RORγt in the mouse model were also negatively correlated with DEGs in both clinical cohorts. Independently, the RORγt-sensitive DEGs from the mouse model inversely correlated most strongly with the transcriptome-associated cluster 2 (TAC2) described by Kuo et al. [34] from U-BIOPRED sputum transcriptomic analysis, a neutrophilic patient cluster which is predominantly Th2-low.

These findings appear to be inconsistent with the recently released negative outcome of the phase II clinical study using the anti-IL-23 antibody, Risankizumab, in a broad severe asthma population (https://clinicaltrials.gov/ct2/show/NCT02443298?term=risankizumab&rank=15). However, a sub-population analysis has not yet been performed, so it is unclear whether a sub-population of patients benefited from treatment. Furthermore, RORγt is a broader mechanism-of-action (MoA) compared with IL-23 blockade in terms of maintenance and activation of IL-17 producing leucocytes and extrapolating these findings to a different MoA is perhaps misleading.

In summary, these data confirm that a Th17 severe asthma sub-population exists which is predominantly neutrophilic, however, neutrophils per se may not necessarily drive the clinically associated symptoms of severe asthma, but rather associated cytokines. In naïve mice, both IL-17 and IL-22 are required to elicit AHR and in a mouse pulmonary inflammation model, RORγt inhibition that inhibits both IL-17 and IL-22 improved lung function and blocked inflammatory mediator release, but this could not be achieved by IL-17 blockade alone. Furthermore, deregulation of RORγt-sensitive genes in mice correlates negatively with DEGs found in bronchial biopsies from severe asthma patients. Taken together, these data suggest that inhibition of RORγt may benefit a sub-population of severe asthma patients to a greater extent than blockade of Il-17 alone.

## Supplementary Information

**Additional file 1: Figure S1.** Female Balb/c mice were senitised with house dust mite (HDM) in complete Freunds adjuvant intraperitoneally on days 0 and 1 and subsequently challenged intratracheally with saline (N = 6) or HDM in saline (N = 8) on days 14 and 15. BIX119 was dosed twice daily on days 14 and 15 by oral gavage. Mice were sacrificed on day 16 and airway eosinophils (a), IL-4 (b) and IL-5 (c) were measured.

## Data Availability

The datasets used and/or analysed during the current study are available from the corresponding author on reasonable request.
