# Supplementary figures and images for: RORγt inhibitors block both IL-17 and IL-22 conferring a potential advantage over anti-IL-17 alone to treat severe asthma
Source: Respir Res. 2021 May 22;22:158. doi: 10.1186/s12931-021-01743-7 (PMC8141258; doi:10.1186/s12931-021-01743-7)

## Slide 1
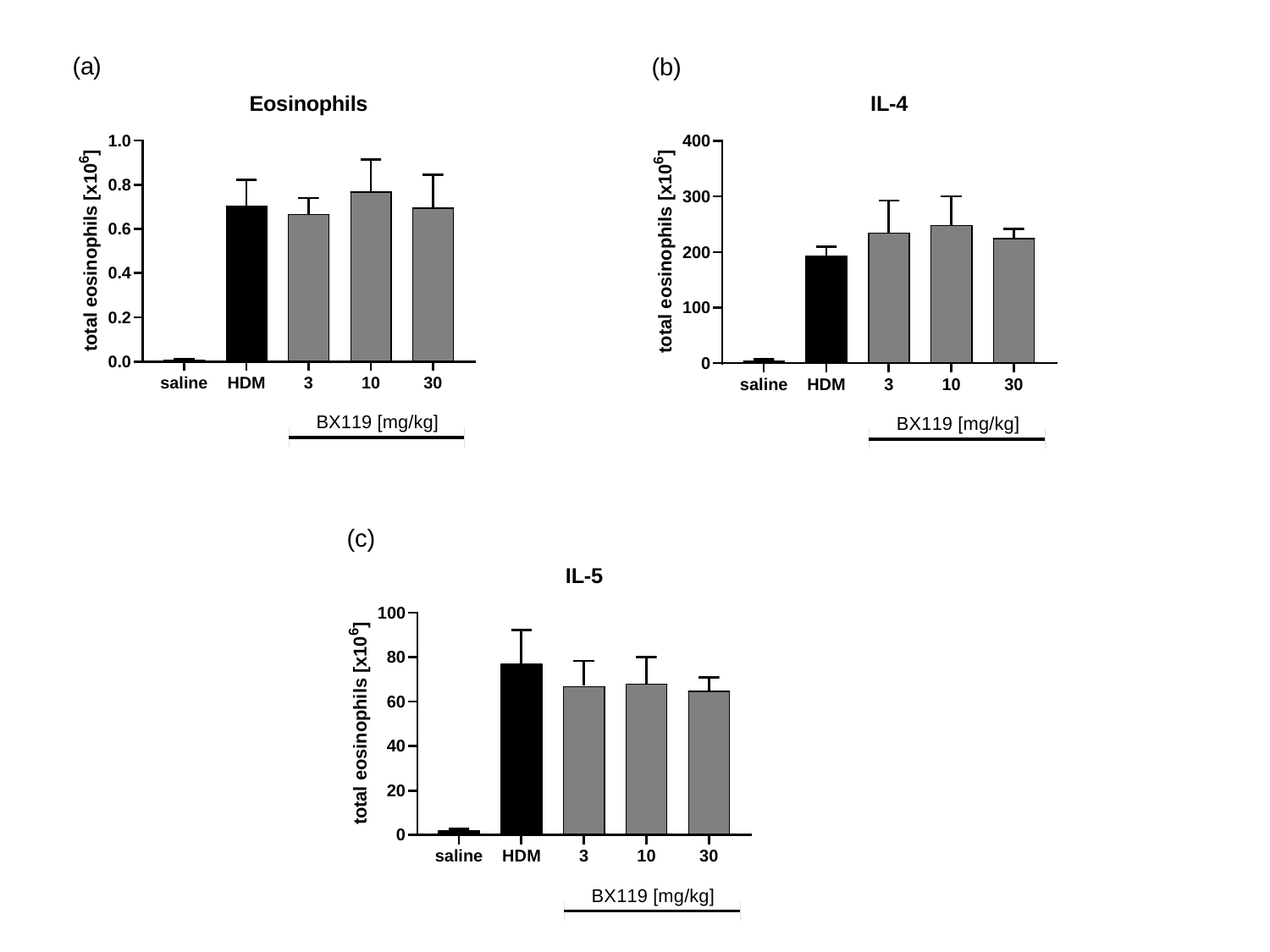

Supplement: Supplementary file 1 — Additional file 1: Figure S1. Female Balb/c mice were senitised with house dust mite (HDM) in complete Freunds adjuvant intraperitoneally on days 0 and 1 and subsequently challenged intratracheally with saline (N = 6) or HDM in saline (N = 8) on days 14 and 15. BIX119 was dosed twice daily on days 14 and 15 by oral gavage. Mice were sacrificed on day 16 and airway eosinophils (a), IL-4 (b) and IL-5 (c) were measured. [file 12931_2021_1743_MOESM1_ESM.pptx]
